# Extreme mortality during a historical measles outbreak on Rotuma is consistent with measles immunosuppression

**DOI:** 10.1017/S095026882400075X

**Published:** 2024-05-13

**Authors:** Susie Cant, G. Dennis Shanks, Matt J. Keeling, Bridget S. Penman

**Affiliations:** 1 Zeeman Institute for Systems Biology and Infectious Disease Epidemiology Research, University of Warwick, Coventry, UK; 2 Mathematics Institute, University of Warwick, Coventry, UK; 3 School of Public Health, University of Queensland, Herston, QLD, Australia; 4 School of Life Sciences, University of Warwick, Coventry, UK

**Keywords:** historical, mathematical modelling, measles (rubeola), Pacific Island, Rotuma

## Abstract

Until the early twentieth century, populations on many Pacific Islands had never experienced measles. As travel to the Pacific Islands by Europeans became more common, the arrival of measles and other pathogens had devastating consequences. In 1911, Rotuma in Fiji was hit by a measles epidemic, which killed 13% of the island population. Detailed records show two mortality peaks, with individuals reported as dying solely from measles in the first and from measles and diarrhoea in the second. Measles is known to disrupt immune system function. Here, we investigate whether the pattern of mortality on Rotuma in 1911 was a consequence of the immunosuppressive effects of measles. We use a compartmental model to simulate measles infection and immunosuppression. Whilst immunosuppressed, we assume that individuals are vulnerable to dysfunctional reactions triggered by either (i) a newly introduced infectious agent arriving at the same time as measles or (ii) microbes already present in the population in a pre-existing equilibrium state. We show that both forms of the immunosuppression model provide a plausible fit to the data and that the inclusion of immunosuppression in the model leads to more realistic estimates of measles epidemiological parameters than when immunosuppression is not included.

## Introduction

Measles is a highly contagious respiratory virus. Sometimes, measles infection can lead to lethal complications including pneumonia, encephalitis, and severe diarrhoea [1, 2]. Measles infection also causes a period of immunosuppression, in which an individual becomes more susceptible to secondary infections and additional complications [3–6]. Since the introduction of a vaccine in the mid-twentieth century, measles deaths have declined significantly, although measles still remains a serious health risk [7]. Pre-vaccine, measles was endemic in most large countries whose population was above the critical community size (300,000–500,000) required to sustain the disease [8].

Many Pacific Island populations first experienced measles in the late nineteenth and early twentieth centuries as a consequence of contact with Europeans. Measles and other pathogens, such as smallpox and dysentery, caused devastating outbreaks across many islands, with far higher measles mortality rates than typically observed in contemporary, well-connected larger populations [9, 10]. Whilst measles outbreaks continue to occur on Pacific Islands up to the present day, including the recent severe outbreak in Samoa in 2019 [7, 11], they no longer display the extreme lethality of the first-contact outbreaks. Instead, they exhibit mortality rates similar to those seen globally [12]. Two explanations have so far been suggested for this phenomenon. The first is that Pacific Island populations (and other isolated populations) were genetically susceptible to severe measles when the pathogen first arrived. The second hypothesis is that repeated exposure to respiratory pathogens (as experienced by those living in large, well-connected populations) builds up broadly protective immune responses, which reduce individual infection mortality from measles, although measles remains a serious infection [9]. Pacific Islanders had little such exposure prior to contact with Europeans, but now they have the same exposure as the rest of the world. Patterns of mortality in US army recruits suggest that epidemiological isolation can contribute to greater measles severity [13], and previous modelling work suggests that an immunological transition is a plausible explanation for the pattern of historical Pacific Island infection mortality [10].

Rotuma is a Fijian island located 646 km north of the capital, Suva. Although most of Fiji experienced measles in a large outbreak in 1875 [14], Rotuma’s distance from other Fijian islands meant that it did not experience measles until 1911 [15]. Due to the 1875 measles outbreak, a medical officer was stationed on Rotuma to check for infectious diseases among those entering the island. Unfortunately, they were absent on 29 January 1911 when two people infected with measles landed on Rotuma [16]. Nearly 13% of the population of the island died in the subsequent epidemic [9]. American Samoa and Guam also experienced measles outbreaks in 1911 and 1913, respectively, although with lower mortality, potentially because these islands had experienced measles outbreaks before [17, 18]. Across all these outbreaks in the Pacific Islands at the time, a high percentage of those with measles infections also suffered extreme gastrointestinal complications [19]. Other high-lethality measles outbreaks in the early twentieth century not on Pacific Islands did not involve similar gastrointestinal complications, but rather involved pulmonary complications. Such pulmonary complications occurred during extreme measles mortality in Boer War concentration camps [20] and as a result of measles and streptococcal coinfection in a measles outbreak among US soldiers in 1917–1918 [21].

As noted previously, measles infection disrupts immune system function [3–6]. Here, we hypothesize that the extreme gastrointestinal complications observed during measles outbreaks on Pacific Islands were specifically due to the immunosuppressive effects of measles. To explore this hypothesis, we model three possible scenarios.

In the first and simplest scenario (model 1), we assume that acute measles infection, with no involvement of any other infectious agent, could lead to all deaths where ‘measles’ was listed as a cause. In the second scenario (model 2), we assume that an additional (non-measles) microbe was brought to Rotuma at the same time as measles, and infection with this agent could cause lethal gastrointestinal disease in those experiencing measles immunosuppression after their initial measles infection. We model this second infectious agent as having susceptible–infectious–recovered (SIR) dynamics because we are assuming that it is also a novel infectious agent, never before seen on Rotuma; given this was a novel introduction, the difference between SIR and alternative formulations is likely to be small. In the third scenario (model 3), we assume that measles immunosuppression triggered dysfunctional immunological reactions to otherwise benign microbes within the Rotuman population (i.e. communicable agents which were already present on the island in an equilibrium state, perhaps gut microbes). For this scenario, we model the secondary infectious agent as having susceptible–infectious–susceptible (SIS) dynamics, because (i) we considered microbes with SIR dynamics to be less likely to persist in very small populations and (ii) SIS dynamics would be the best way to simulate a component of the gut microbiome, the likely trigger for the gastrointestinal complications. We use ordinary differential equation models to capture all of the aforementioned scenarios, fit each model to the available data from the Rotuman 1911 measles outbreak, and assess the plausibility of the resulting fitted parameter values.

## Methods

### Models 1–3

**Figure 1.**
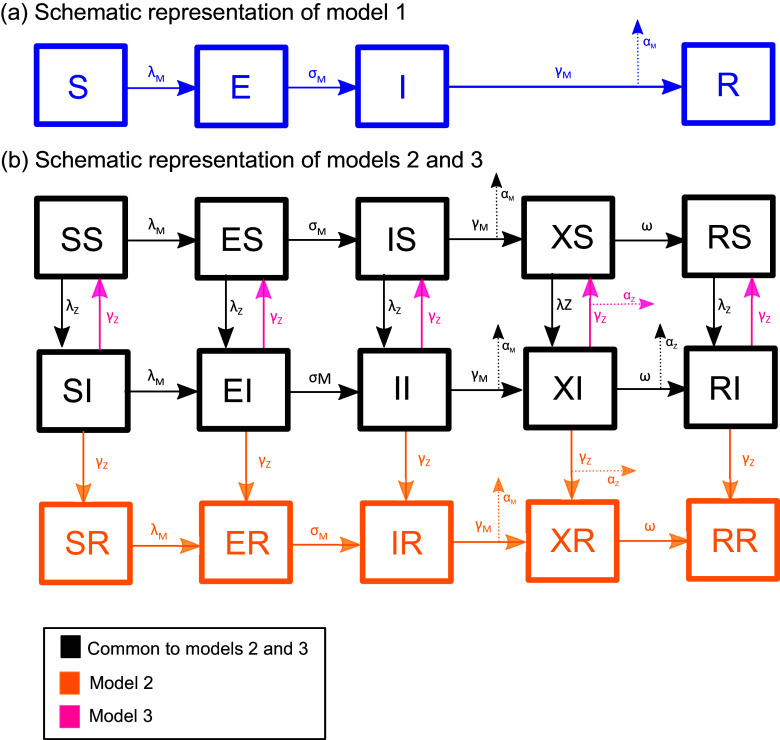
Schematic diagram of compartmental models 1–3. Model 1 is shown in panel (a), and models 2 and 3 are shown in panel (b). Each box represents a different state in which an individual can exist (see Methods for how these are defined). Solid arrows represent the rates of transition between different states. Dotted arrows represent losses due to infectious disease mortality. Specifically, the symbols α_M_ and α_Z_ represent proportions of those who would have transitioned between two states, but in fact died from acute measles (α_M_) or infection whilst immunosuppressed (α_Z_). Definitions of all rate symbols used are given in Table 1, with the exception of the symbols λ_M_ and λ_Z_, which represent the force of infection with measles and the secondary infectious agent, respectively, and are defined with the model equations in the Supplementary Material.

All three of our models are compartmental models in which measles infects a population of size *N*, with or without an additional infectious agent present. Given the timescale involved, we ignore births and immigration. Model 1 is an SEIR model in which hosts may be susceptible to measles (S), exposed to measles (E), infectious with measles (I), or recovered/immune to measles (R). Models 2 and 3 extend the SEIR model to include an immunosuppressed state – thus, hosts could be susceptible (S), exposed (E), infectious (I), immunosuppressed (X), or recovered/immune (R). We modelled the dynamics of the second infectious agent as susceptible, infectious then recovered/immune (SIR) in model 2 or susceptible, infectious then susceptible again (SIS) in model 3. For models 2 and 3, we summarized the state of each host with respect to measles and the second infectious agent by stating their position in each set of possible conditions. ‘IS’ therefore refers to a host who is infectious with measles and susceptible to the second infectious agent; ‘RI’ represents a host who is immune to measles but infectious with the second infectious agent, and so on. Models 2 and 3 are illustrated schematically in Figure 1, and equations for all three models are given in the Supplementary Material.

### Parameter selection and initial conditions

All model parameters are listed in Table 1. For all but three parameters, we tested a range of possible values to determine which provided the best fit to the data. We always fixed the value of the average latent period of measles (1/σ_M_) and the average infectious period of measles (1/γ_M_). It is reasonable to assume that the measles virus which infects people today is unchanged from that which existed at the beginning of the twentieth century. A measles generation time of approximately 12 days was estimated by Hope Simpson from a detailed study of measles in households [22]. A statistical analysis [23] of Hope Simpson’s detailed data set estimated a length of the latent period to be 7.63 days and the infectious period to be 7.05 days. We therefore took the average length of time in the exposed class to be 8 days and the average duration of the infectious period to be 7 days.

**Table 1.** Parameters used in models

Parameter	Description	Value used
β_M_	Measles transmission parameter, such that in the model framework used here the basic reproduction number of measles (R_0M_) is equal to 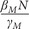	Values between 0.00006 and 0.0005 explored in least–squares analyses. Also fitted to the data using MCMC with a uniformly distributed prior (minimum and maximum values as given above)
σ_M_	1/average duration of latent period of measles (rate of transitioning from the measles exposed class to the measles infectious class)	1/8 days^−1^
γ_M_	1/average period of infectiousness with measles (rate of transitioning from the measles infectious class to the measles immunosuppressed class)	1/7 days^−1^
ω	1/average period of measles immunosuppression (rate of transitioning from the measles immunosuppressed class to the measles immune class)	Values between 0.01 and 0.14 days^−1^ explored in least–squares analyses. Also fitted to the data using MCMC with a uniformly distributed prior (minimum and maximum values as given above)
β_Z_	Second infectious agent transmission parameter such that in the model framework used here the basic reproduction number of the second infectious agent is equal to 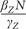	Values between 0.00006 and 0.0005 explored in least–squares analyses; fixed at a value of 0.000096 for the MCMC analysis (equivalent to R_0Z_ = 1.61)
γ_Z_	1/average period of infectiousness with second infectious agent (rate of transitioning from the infectious class to the susceptible (model 1) or recovered (model 2) class)	1/7 days^−1^
α_M_	Case fatality rate of measles	Values between 0 and 0.5 explored in least–squares analyses. Fitted to the data using MCMC with a uniformly distributed prior with minimum and maximum values of 0 and 1. We used a wider range for the prior in the MCMC analysis than was used in the least–squares analysis, because in the least–squares analysis, it seemed as though the best fits were achieved when α_Z_ took values that were close to the maximum possible value in the least–squares analysis (0.5) – see Supplementary Figures S3 and S4
α_Z_	Case fatality rate for those infected with secondary infectious agent whilst simultaneously in immunosuppressed class	

We also fixed the average duration of infections with the secondary infectious agent (1/*γ_Z_*). This was a simplifying assumption to reduce the number of parameters we had to attempt to fit with limited data. We fixed *γ_Z_* at a value which implied an average duration of infectiousness of 1 week (7 days), which is not unreasonable for many infectious agents. The transmission parameter of the secondary infectious agent (β_z_) could vary; thus, the basic reproduction number of the secondary infectious agent (R_0Z_) was still being fitted in our analysis, despite this simplifying assumption.

The initial conditions of the model were based on the population size of Rotuma in early 1911 (N = 2,401, made up of 2,399 individuals living on the island, plus 2 who were known to have brought measles to the island at time 0; we do not attempt to account for any other individuals arriving by boat). It is not clear from the historical record whether the two individuals who arrived carrying measles were already infectious (i.e. in the infectious class) or shortly to become infectious (i.e. in the exposed class) at time 0. We therefore tested both possibilities: we placed two individuals in class E for model 1 and class ES for models 2 and 3 to simulate the individuals arriving in the exposed class, or we placed two individuals in class I for model 1 and class IS for models 2 and 3 to simulate the individuals arriving in the infectious class. For model 2, we assumed there to be one individual in class SI at time 0 (measles susceptible, infectious with secondary infectious agent). For model 3, we set the number of individuals in class SI at time 0 to be such that the proportion of individuals in class SI at time 0 was equal to the equilibrium for the secondary infectious agent 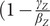 ).

### Mortality data

Detailed census data for Rotuma covering the 1911 period are available, including the cause of death. These mortality data have previously been published [9]. For our analysis, we focus on deaths where measles was listed as one of the causes. We group the deaths into two classes: measles without gastrointestinal complications (i.e. where measles is named as a cause of death, but diarrhoea or ileocolitis is not named) and measles with gastrointestinal complications (i.e. where the cause of death is given as measles with diarrhoea or measles with ileocolitis). In some cases, measles is listed alongside a non-gastrointestinal complication. We include all these deaths in the ‘measles without gastrointestinal complication’ category, with the exception of any which included tuberculosis (phthisis) as a cause. Measles with phthisis deaths typically occurred months after the initial epidemic. These were likely due to the reactivation of tuberculosis by measles immunosuppression, but we did not include this process in our model; hence, it was simplest not to include these deaths.

### Model fitting

To fit each model to the data, we considered deaths occurring after measles infection, with or without complications due to immunosuppression. For model 1, the only modelled deaths were those which occurred on leaving class I and there was no distinction between measles with and without gastrointestinal complications. When we included a secondary infectious agent (models 2 and 3), all modelled deaths occurring upon leaving classes IS, II, or IR were counted as ‘measles without gastrointestinal complications’ deaths and all modelled deaths occurring upon leaving class XI were counted as ‘measles with gastrointestinal complications’ deaths.

We employed two approaches to explore which parameter values provided the best fit of each model to the data: least-squares and Bayesian Markov chain Monte Carlo (MCMC) analyses.

In the least-squares approach, we recorded the sum of the squared deviations between the daily mortality numbers reported on Rotuma and those predicted by our models for different sets of values of β_M_, β_Z_, α_M_, α_Z_, and ω. Values of β_M_, β_Z_, α_M_, α_Z_, and ω were sampled using Latin hypercube sampling from the parameter space indicated in Table 1. Our first analysis considered the total number of deaths due to measles each day (i.e. the sum of ‘measles without gastrointestinal complications’ and ‘measles with gastrointestinal complications’). For this analysis, we sampled ten million different sets of parameters and fitted models 1, 2, and 3.

Our second analysis split up the daily measles deaths into ‘measles without gastrointestinal complications’ and ‘measles with gastrointestinal complications’. We tested six million different sets of parameters. Only models 2 and 3 could be fitted in the second analysis because only these models make separate predictions for measles deaths with and without gastrointestinal complications.

For the MCMC approach, we used the slice sampling MCMC algorithm [24] implemented in MATLAB with a Poisson likelihood. We fitted model 1 to the total daily measles deaths (the sum of ‘measles without gastrointestinal complications’ and ‘measles with gastrointestinal complications’ each day), and we fitted models 2 and 3 to two values per day (‘measles without gastrointestinal complications’ and ‘measles with gastrointestinal complications’). We used uninformative uniform priors (given in Table 1) for β_M_, α_M_, α_Z_, and ω, but fixed the value of β_Z_ based on the results of our least-squares analysis as described in the Results. Effective sample size (ESS) and 95% highest posterior density intervals were obtained using Tracer [25]. We ran the MCMC for long enough that the ESS was >200 for all estimated parameters.

For both approaches (least squares and MCMC), we sampled the value of ω (the rate of leaving the immunosuppressed compartment) as part of the analysis. However, for ease of interpretation, in our figures and results, we present the value of 1/ω (the mean duration of immunosuppression).

## Results

### All three models can reproduce the broad pattern of total measles mortality

**Figure 2.**
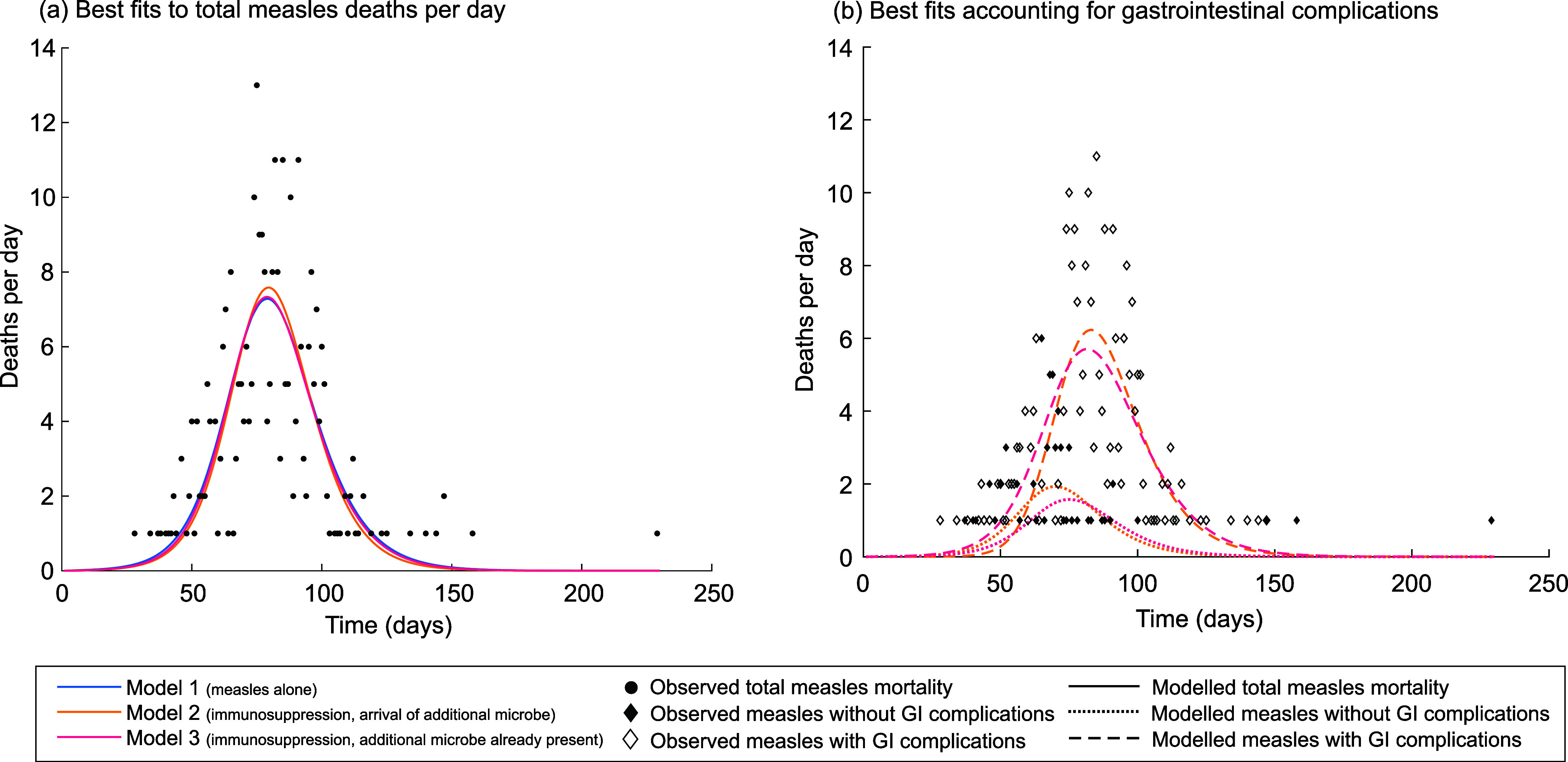
Least-squares fitting of mortality patterns during the 1911 measles outbreak on Rotuma. Panel (a) illustrates the best-fitting mortality time series generated by each of models 1–3 using least-squares fitting when the models were fitted to the total number of measles deaths per day. Panel (b) illustrates the best-fitting mortality time series for models 2 and 3 using least-squares fitting when the models were fitted to the pattern of measles deaths with and without gastrointestinal complications. In both panels (a) and (b), the two individuals who brought measles to Rotuma were assumed to be in the exposed class. The equivalent results when the two individuals were assumed to be in the infectious class are shown in Supplementary Figure S2.

Our simplest model (model 1) assumes that all measles deaths on Rotuma occurred due to acute measles infection. To compare this model with our two immunosuppression models (models 2 and 3), we used the least-squares approach described in the methods, fitting to the total number of measles deaths recorded each day. Model 2, which assumes immunosuppression and the arrival of a second novel microbe alongside measles, achieved the best fit (lowest sum of squares) out of all three models (Supplementary Table S1). However, visual inspection of the fitted dynamics reveals little difference between the models in terms of their ability to capture the overall epidemic curve (Figure 2a).

As noted in the introduction, measles deaths could be split into those with and without gastrointestinal complications. We fitted models 2 and 3 to this more complex data set using the least-squares approach. Both models 2 and 3 could reproduce the patterns of measles deaths with and without gastrointestinal complications implied by the Rotuma data (Figure 2b). Again, model 2 provided the slightly better fit (Supplementary Table S1) and was better able to allow the wave of ‘without gastrointestinal complications’ deaths to peak earlier than the wave with such complications (Figure 2b).

Placing the two individuals who brought measles to Rotuma in the infectious or exposed class at time 0 made essentially no difference to the fits that could be achieved (Supplementary Table S1). Throughout the main text, we present results in which we assume that those two individuals were in the exposed class. We present equivalent results assuming that they were in the infectious class in the Supplementary Material.

### Allowing measles immunosuppression to account for mortality on Rotuma in 1911 leads to higher estimates of the R_0_ of measles and lower estimates of the case fatality rate of acute measles than when immunosuppression is not included

Supplementary Figures S1 and S2 display combinations of parameter values which were associated with plausible fits to the data in our least-squares fitting of models 2 and 3 (see Supplementary Methods for how this plausible fit was defined). For model 2, R_0Z_ (the basic reproduction number of the secondary infectious agent) can only fall within a very narrow range of values: 1.55 to 1.73 if the two individuals who brought measles to Rotuma are assumed to be in the exposed class when they arrived at day 0 (and the very similar range of 1.53 to 1.77 if these individuals are assumed to be in the infectious class at day 0). For model 3, R_0Z_ can take a wide range of values, but these are highly correlated with the case fatality rate of individuals experiencing infections during the period of measles immunosuppression (α_Z_).

It is clear from the least-squares analysis that a range of different parameter sets could be consistent with the Rotuman pattern, and the data are insufficient to determine a single best-fitting scenario. Nevertheless, if we fix a value for the reproductive number of the secondary infectious agent (R_0Z_), we can use MCMC to estimate the posterior distribution for the other parameters of the model for *that* possible value of R_0Z_. We fixed R_0Z_ at a value of 1.61 by fixing β_Z_ = 0.000096. This value allowed good fits for model 2 in the least-squares analysis. As noted above, there was no limitation on the possible values which allow good fits for model 3, and in the Supplementary Material, we show the impact of applying two other values of β_Z_ (0.0000655 to give R_0Z_ = 1.1 and 0.000119 to give R_0Z_ = 2).

Having fixed R_0Z_, we used MCMC to determine posterior distributions for β_M_, α_M_, α_Z_, and ω for models 2 and 3 and for β_M_ and α_M_ for model 1.

**Figure 3.**
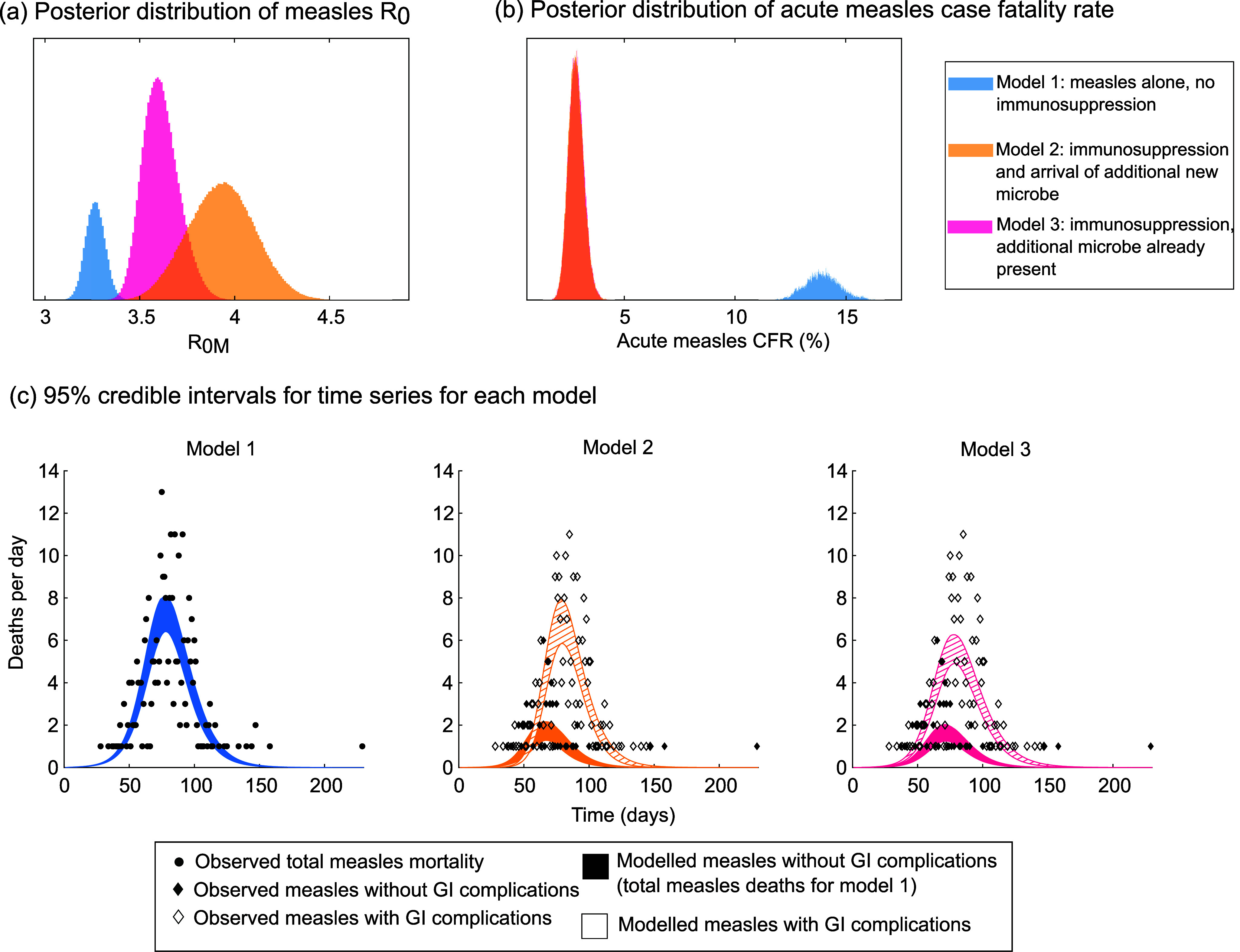
Estimates of measles R_0_ and acute measles case fatality rate in models with and without immunosuppression. These results are for the scenario where the two individuals who brought measles to Rotuma were both in the exposed (not yet infectious) class at time = 0. Panels (a) and (b) illustrate posterior distributions for the basic reproduction number of measles (a) and the case fatality rate of acute measles (b), obtained using MCMC as described in the Methods. In panel (b), the estimates for models 2 and 3 are so similar that the distributions overlap. Panel (c) illustrates time series for each of the three scenarios explored: (i) model 1 , in which we do not separate deaths caused by measles alone from deaths associated with both measles and gastrointestinal complications; (ii) the model 2 immunosuppression scenario, in which measles enters the population at the same time as a second novel microbe; and (iii) the model 3 immunosuppression scenario, in which measles disrupts an existing microbial equilibrium on Rotuma. The 95% credible intervals for the model outputs were obtained by sampling 2000 different parameter sets from the joint posterior distribution of all parameters, running the model with each parameter set, recording the range of numbers of deaths per day observed at each time point for all those different parameter values, and then truncating that range by 2.5% from the top and 2.5% from the bottom for each time point.

Figure 3 illustrates the posterior distributions of measles epidemiological parameters obtained from our MCMC fitting. Models which include measles immunosuppression (i.e. model 2 or model 3) are consistently associated with higher values for the R_0_ of measles than model 1. When we assume that the two individuals who brought measles to Rotuma started out in the exposed class at time 0, measles R_0_ is estimated to be 3.27 (3.17, 3.37) for model 1, 3.93 (3.59, 4.27) for model 2, and 3.61 (3.44, 3.80) for model 3; thus, model 2 is associated with the highest value for measles R_0_.

Models 2 and 3 also generate much lower estimates of the case fatality rate of acute measles infection than model 1. When we assume that the two individuals who brought measles to Rotuma started out in the exposed class at time 0, the case fatality rate is 0.1394 (0.1244, 0.1557) for model 1, but the much lower 0.0281 (0.0215, 0.0350) for model 2 and 0.0282 (0.0216, 0.0351) for model 3.

Supplementary Figures S5 and S6 illustrate that assuming that the two individuals who brought measles to Rotuma were in the infectious class rather than the exposed class leads to slightly lower estimates of R_0_ than those given above and very similar estimates of the acute measles case fatality rate. Choosing different values of R_0Z_ for model 3 has limited impact on the estimates achieved (Supplementary Figures S5 and S6).

### The Rotuman data are consistent with a period of risky immune dysregulation lasting up to 3 weeks

**Figure 4.**
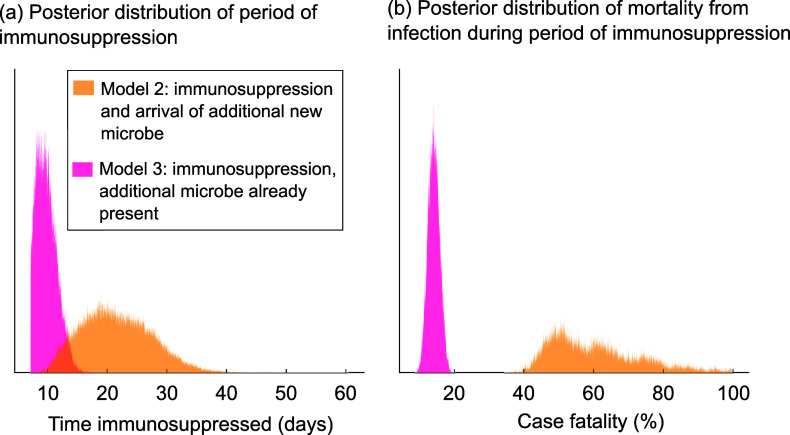
Duration of period of immunosuppression and mortality whilst immunosuppressed. These results are for the scenario where the two individuals who brought measles to Rotuma were both in the exposed (not yet infectious) class at time = 0. Panels (a) and (b) illustrate posterior distributions for the average duration of the period of immunosuppression following measles infection (a) and the case fatality rate for those infected with a second agent during that period (b), using model 2 or model 3 (different colours, as indicated in the key).

Model 2, in which we assume that measles arrives alongside a second infectious agent, is consistently associated with a longer period of immunosuppression than model 3 (Figure 4a and Supplementary Figure S6a). When we assume that the two individuals who brought measles to Rotuma started out in the exposed class at day 0, the mean period of immunosuppression is 22 days (11, 33 days) for model 2 and 10 days (7, 13 days) for model 3.

Both models 2 and 3 also need to invoke a very high case fatality rate for those who become infected with the secondary infectious agent during the period of immunosuppression. When we assume that the two individuals who brought measles to Rotuma started out in the exposed class at day 0, the case fatality rate for infection whilst immunosuppressed is 0.604 (0.418, 0.880) for model 2 and 0.1422 (0.111, 0.174) for model 3.

Choosing a different value for R_0Z_ affects the estimated duration of immunosuppression and case fatality rate whilst immunosuppressed for model 3, with a lower value of R_0Z_ associated with a longer period of immunosuppression and a higher case fatality rate for infection whilst immunosuppressed (Supplementary Figure S6).

## Discussion

There is every reason to suppose measles caused immunosuppression on Rotuma in 1911. Measles infection is known to have this effect [26], and reports of reactivated tuberculosis infections at the time of the measles outbreak are consistent with immunosuppression occurring [9]. Our key finding is that there is no need to invoke an especially high death rate from acute measles on Rotuma in 1911, if the Rotuman population was susceptible to lethal gastrointestinal complications during a period of immunosuppression following measles infection (Figure 3b). This result adds to the growing argument that devastatingly lethal first-contact epidemics need not have been due to any particular genetic susceptibility of a previously isolated human population [10]. Instead, they can be understood in terms of epidemiological phenomena such as lack of prior immune exposure, immunosuppression, and coinfection.

All of our models (with or without immunosuppression) could convincingly reproduce the overall wave of measles deaths (Figure 2). Thus, at the only level at which all three models could be compared, there was little to choose between them. The true difference between the models with and without immunosuppression becomes apparent when we consider how each model estimates the epidemiological parameters of measles (Figures 3 and 4). As discussed below, the values given by models 2 and 3 are far more convincing and in keeping with modern estimates for measles than the values given by model 1. Based on this, we assert that immunosuppression was indeed important in generating the measles mortality pattern seen on Rotuma in 1911.

The basic reproductive number of measles (R_0_) depends on a range of factors, including population density and cultural practises, as well as the intrinsic properties of the virus. The estimated measles R_0_ values that we obtain for Rotuma in 1911 of up to 3.93 (3.59, 4.27) are towards the lower end of what has been previously reported for measles, for which R_0_ is often assumed to be at least 12 [27]. A systematic review of the R_0_ of measles in a range of non-virgin-soil settings found that out of 58 different estimates, the majority (52) were greater than 6. However, in three cases the R_0_ value of measles was found to be between 4.1 and 6; in one case the value was estimated to be between 2.1 and 4, and in one case it was lower than 2.1 [27]. Broutin et al. [28] estimated the R_0_ value of measles to be 4.6 in Niakhar, Senegal, which ‘contains 30 villages of sizes ranging from 50 to 3,000 inhabitants…The compound, representing the smallest structure of the zone, corresponds to a group of houses where extended families live, in one or several households’. A rural setting such as this may be more relevant to Rotuma than others in which measles R_0_ estimates have been obtained. Broutin’s estimate of measles R_0_ = 4.6 is similar to the highest estimate of R_0_ that we obtained for the Rotuman data: 3.93 (3.59, 4.27) for model 2 when the two individuals who brought measles to Rotuma were in the exposed class on day 0. Another important aspect is that higher values of measles R_0_ (e.g. R_0_ = 16–18 for England and Wales [29]) are generally estimated in urban endemic settings where the transmission is concentrated in closely mixing schoolchildren. In contrast, transmission in Rotuman is spread across the entire population, which is mainly in small rural communities.

A study of measles fatality in low- and middle-income countries between 1980 and 2016 found the case fatality rate for measles in the community to be 2·4% (0·0–9·8) for low-income countries and 1·4% (0·0–5·8) for lower-middle-income countries [30]. A detailed analysis of measles mortality in rural Bangladesh found the case fatality rates for measles to vary between 0 and 0.018 when the children concerned had not been born during a period of famine and 0.038 when the majority of the children concerned had been born during a period of famine [31] (a fascinating insight into the complexity of measles mortality). The overall pattern of these case fatality rates for low-income/rural settings is consistent with our estimates for the acute case fatality rate of measles on Rotuma of 0.0281 (0.0215, 0.0350) for model 2 and 0.0282 (0.0216, 0.0351) for model 3 (remembering that both models 2 and 3 assume that the gastrointestinal complications are a consequence of immunosuppression under a unique set of circumstances and do not count them as direct measles mortality). The basic epidemiological features of measles on Rotuma in 1911, for our immunosuppression models, thus seem consistent with those seen around the world in comparable modern settings.

Our two immunosuppression models are based on different biological concepts of what could trigger gastrointestinal complications during a period of immunosuppression. Model 2, in which measles arrived on Rotuma alongside a second infectious agent, is conceptually simple. There could have existed a viral or bacterial agent on board the ship which arrived on Rotuma in 1911, which, like measles, the Rotuman population had never encountered before. However, this scenario lacks parsimony in that we need to suppose the existence of a second novel pathogen alongside measles to explain the Rotuman pattern of mortality. Model 3 supposes that infectious agents, shared among the Rotuman population in a state of dynamic equilibrium before measles arrived, determined people’s susceptibility to gastrointestinal complications when immunosuppressed by measles. The most likely identity of these agents is gut microbes. We know that the gut microbiome is dynamic, with members of a household swapping and sharing specific microbial clones [32]. The gut microbiomes of industrialized societies, subsistence farmers, and hunter–gatherer societies exhibit distinct features [33, 34], implying that the changes in the human gut microbiome are associated with transitions in human lifestyle. A previously suggested explanation for the extreme mortality of early Pacific Island dysentery epidemics is that the diverse gut microbiomes of highly isolated human populations exist in a state of equilibrium with the immune system, the disruption of which can result in extreme gastrointestinal disease [35]. For measles, immunosuppression (rather than dysentery directly disrupting the gut microbiome) would be the driver behind such dysregulation, but the riskiness of a diverse Pacific Islander gut microbiome in the face of a novel infection could be a common feature of extreme infection mortality in both historical measles and dysentery outbreaks. The fact that we no longer see such extreme gastrointestinal complications following measles outbreaks today could reflect the microbial transition of Pacific Islander societies, such that their gut microbiomes are now more similar to those of all industrialized societies.

A matched cohort study found that the children who experienced measles infection in the UK between 1990 and 2014 were more susceptible to non-measles infectious disease than controls who did not experience measles [36]. This effect lasted up to 5 years following measles infection, but the biggest differences between the measles-infected children and the controls occurred in the first month following measles infection. For our immunosuppression modelling, the duration of risky immune dysregulation on Rotuma was estimated at approximately 20 days for model 2 and approximately 10 days for model 3 (but if the secondary infectious agent has an R_0_ value of 1.1 in model 3, the duration of immunosuppression is increased to up to 20 days – see Supplementary Figure S6).

The widespread availability of measles vaccination is a pillar of global health, but vaccine hesitancy and scepticism undermine the uptake of this lifesaving intervention [37]. Modern populations are only protected against the impact of measles if their vaccination rates are sufficiently high. In 2019, the measles vaccine rate was only 31% in Samoa, although thankfully higher in other Pacific Island populations [38]. Measles vaccination rates had generally been declining on Samoa from 2014 [11], but trust in the measles vaccine was especially shaken in Samoa due to a tragic human error in vaccine delivery in 2018 [39]. Following 2019’s especially low measles vaccination rate, there was a measles outbreak in Samoa in 2019 causing 5,707 cases, 1868 hospitalizations, and 83 deaths [11]. The ongoing public health impact of this outbreak, in terms of the impact of measles on the immune systems of those affected, remains to be seen.

The modelling that we present here offers several new perspectives on the 1911 Rotuman measles outbreak. We demonstrated that once immunosuppression is accounted for, the epidemiological properties of measles itself appear consistent with comparable modern settings. We introduced two alternative scenarios for what could have driven extreme gastrointestinal complications: the introduction of a second novel pathogen or the disruption of an existing gut microbe equilibrium. Whilst the specific circumstances of Rotuma’s isolation in 1911 are never going to be repeated, the 1911 measles outbreak on Rotuma serves to remind us of the potentially lethal impact of measles on the human immune system. A better understanding of this, and all other risks of measles, needs to cut across misinformation and vaccine hesitancy.

## Supporting information

Cant et al. supplementary material Cant et al. supplementary material

## Data Availability

The historical mortality data used in this paper have been previously published [9]. The novel data introduced by this paper are the models and simulation results, which are fully described within the manuscript.

## Supplementary material

For supplementary material accompanying this paper visit http://doi.org/10.1017/S095026882400075X. click here to view supplementary material
