# Supplementary material for: Extreme mortality during a historical measles outbreak on Rotuma is consistent with measles immunosuppression
Source: Epidemiol Infect. 2024 May 13;152:e85. doi: 10.1017/S095026882400075X (PMC11149033; doi:10.1017/S095026882400075X)
Supplement: Cant et al. supplementary material [file S095026882400075Xsup001.docx]

**Extreme mortality during a historical measles outbreak on Rotuma is consistent with measles immunosuppression: Supplementary Material**

This supplementary material contains:

1. Supplementary Methods (pages 1-3)
2. Supplementary Tables (page 4)
3. Supplementary Figures (pages 5-11)
4. **Supplementary Methods**

**Equations for model 1**

The rates of change of numbers of individuals in the different compartments are as follows:

$$\frac{dS}{dt}=-\lambda_{M}S$$

$$\frac{dE}{dt}= \lambda_{M}S- \sigma E$$

$$\frac{dI}{dt}= \sigma E-\gamma_{M}I$$

$$\frac{dR}{dt}=\gamma_{M}\left( 1-\alpha_{M} \right)I$$

Where $\lambda_{M}$ is the force of infection with measles, and is calculated as follows:

$$\lambda_{M}=\beta_{M}I$$

All other parameters are described in table 1.

**Equations for model 2**

The rates of change of numbers of individuals in the different compartments are as follows:

$$\frac{dSS}{dt}=-(\lambda_{M}+\lambda_{Z})SS$$

$$\frac{dES}{dt}= \lambda_{M}SS-(\sigma+\lambda_{Z})ES$$

$$\frac{dIS}{dt}= \sigma ES-{(\gamma}_{M}+\lambda_{Z})IS$$

$$\frac{dXS}{dt}= \gamma_{M}(1-\alpha_{M})IS-(\omega+\lambda_{Z})XS$$

$$\frac{dRS}{dt}=\omega XS-\lambda_{Z}RS$$

$$\frac{dSI}{dt}=\lambda_{Z}SS-(\lambda_{M}+\gamma_{Z})SI$$

$$\frac{dEI}{dt}= {\lambda_{Z}ES+\lambda}_{M}SI-(\sigma+\gamma_{Z})EI$$

$$\frac{dII}{dt}= \lambda_{Z}IS+\sigma EI-{(\gamma}_{M}+\gamma_{Z})II$$

$$\frac{dXI}{dt}=\lambda_{Z}XS+ \gamma_{M}(1-\alpha_{M})II-(\omega+\gamma_{Z})XI$$

$$\frac{dRI}{dt}=\lambda_{Z}RS+\omega\left( 1-\alpha_{z} \right)XI-\gamma_{Z}RI$$

$$\frac{dSR}{dt}=\gamma_{Z}SI-\lambda_{M}SR$$

$$\frac{dER}{dt}= {\gamma_{Z}EI+\lambda}_{M}SR-\sigma ER$$

$$\frac{dIR}{dt}= \gamma_{Z}II+\sigma ER-\gamma_{M}IR$$

$$\frac{dXR}{dt}=\gamma_{Z}(1-\alpha_{Z})XI+ \gamma_{M}(1-\alpha_{M})IR-\omega XR$$

$$\frac{dRR}{dt}=\gamma_{Z}RI+\omega XR$$

Where $\lambda_{M}$ is the force of infection with measles, and is calculated as follows:

$$\lambda_{M}=\beta_{M}(IS+II+IR)$$

And $\lambda_{Z}$ is the force of infection with the secondary infectious agent, and is calculated as follows:

$$\lambda_{Z}=\beta_{Z}(SI+EI+II+XI+RI)$$

All other parameters are described in table 1.

**Equations for model 3**

$$\frac{dSS}{dt}=\gamma_{Z}SI-(\lambda_{M}+\lambda_{Z})SS$$

$$\frac{dES}{dt}= \gamma_{Z}EI+\lambda_{M}SS-(\sigma+\lambda_{Z})ES$$

$$\frac{dIS}{dt}= \gamma_{Z}II+\sigma ES-{(\gamma}_{M}+\lambda_{Z})IS$$

$$\frac{dXS}{dt}= \gamma_{Z}(1-\alpha_{Z})XI+\gamma_{M}(1-\alpha_{M})IS-(\omega+\lambda_{Z})XS$$

$$\frac{dRS}{dt}=\gamma_{Z}RI+\omega XS-\lambda_{Z}RS$$

$$\frac{dSI}{dt}=\lambda_{Z}SS-(\lambda_{M}+\gamma_{Z})SI$$

$$\frac{dEI}{dt}= {\lambda_{Z}ES+\lambda}_{M}SI-(\sigma+\gamma_{Z})EI$$

$$\frac{dII}{dt}= \lambda_{Z}IS+\sigma EI-{(\gamma}_{M}+\gamma_{Z})II$$

$$\frac{dXI}{dt}=\lambda_{Z}XS+ \gamma_{M}(1-\alpha_{M})II-(\omega+\gamma_{Z})XI$$

$$\frac{dRI}{dt}=\lambda_{Z}RS+\omega\left( 1-\alpha_{z} \right)XI-\gamma_{Z}RI$$

Where $\lambda_{M}$ is the force of infection with measles, and is calculated as follows:

$$\lambda_{M}=\beta_{M}(IS+II)$$

And $\lambda_{Z}$ is the force of infection with the secondary infectious agent, and is calculated as follows:

$$\lambda_{Z}=\beta_{Z}(SI+EI+II+XI+RI)$$

All other parameters are described in table 1.

**Supplementary methods for least squares fitting**

We wanted to use the least squares analysis to explore which parameter combinations were able to reproduce the mortality pattern seen on Rotuma. We deemed a value of a sum of squares of 550 or below to be an acceptable fit when fitting models 2 and 3 to the dataset which separated measles deaths with and without gastrointestinal complications. This was an arbitrary cutoff, based on visual inspection of the pattern achieved. Figure S1 shows the worst fitting dynamics allowed by this cut off. All the parameter combinations shown in figures S3 and S4 achieve fits as good as, or better than, those illustrated in figure S1.

1. **Supplementary Tables**

**Table S1 Sums of squares values for best fits achieved for models 1-3.** As described in the Methods and Results, we conducted two separate least squares analyses. In the first, we fitted models 1, 2 and 3 to the total number of measles-associated deaths per day. Sums of squares from this analysis can be directly compared for all 3 models to determine which model provided the closest fit to the overall mortality data. We also carried out a second least squares analysis, where the measles deaths were separated into those with or without gastrointestinal complications. This analysis could only be conducted for models 2 and 3, because only these two models separated out measles deaths into two different types. Sums of squares from this analysis can be directly compared for models 2 and 3, but it is not meaningful to compare the sums of squares from the second analysis to those achieved in the first.

| **Model** | **Starting conditions** | **Smallest sum of squares achieved, fitting to the total number of measles-associated deaths per day** | **Smallest sum of squares achieved, fitting to measles with gastrointestinal complications and measles without gastrointestinal complications deaths per day separately.** |
| --- | --- | --- | --- |
| Model 1 (measles is the only infectious agent present and immunosuppression is irrelevant) | Two individuals arrive in the infectious class | 405.8 | n/a |
|  | Two individuals arrive in the exposed class | 395.8 | n/a |
| Model 2 (immunosuppression and the arrival of a second infectious agent) | Two individuals arrive in the infectious class | 390.1 | 483.4 |
|  | Two individuals arrive in the exposed class | 389.9 | 483.5 |
| Model 3 (immunosuppression disrupts the equilibrium state of an existing microbe on the island) | Two individuals arrive in the infectious class | 403.0 | 524.6 |
|  | Two individuals arrive in the exposed class | 394.6 | 512.0 |

1. **Supplementary Figures**

**
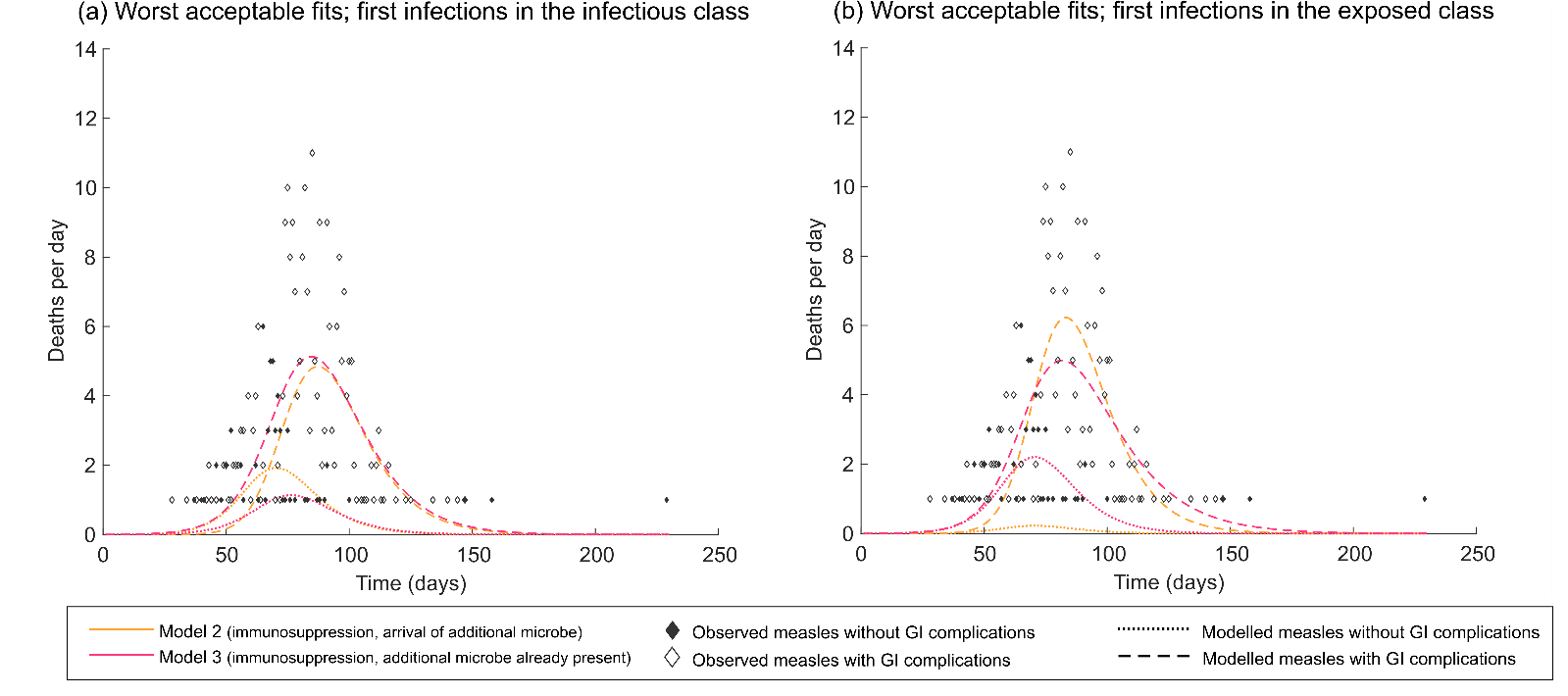
**

**Figure S1: Examples of the worst fitting dynamics that we deemed acceptable for either model 2 or model 3.** This figure illustrates time series for the worst fitting sets of parameters that we deemed acceptable in our least squares analysis of models 2 and 3, where measles deaths were split into two different classes. Panels (a) and (b) show different starting conditions. The sums of squares are: 549.6 for model 2 and 550.0 for model 3 in panel (a) and 549.8 for model 2 and 550.0 for model 3 in panel (b). Only parameter combinations which generated fits of < 550 (and hence were as good as the fits above, or better) are included in figures S3 and S4.

**
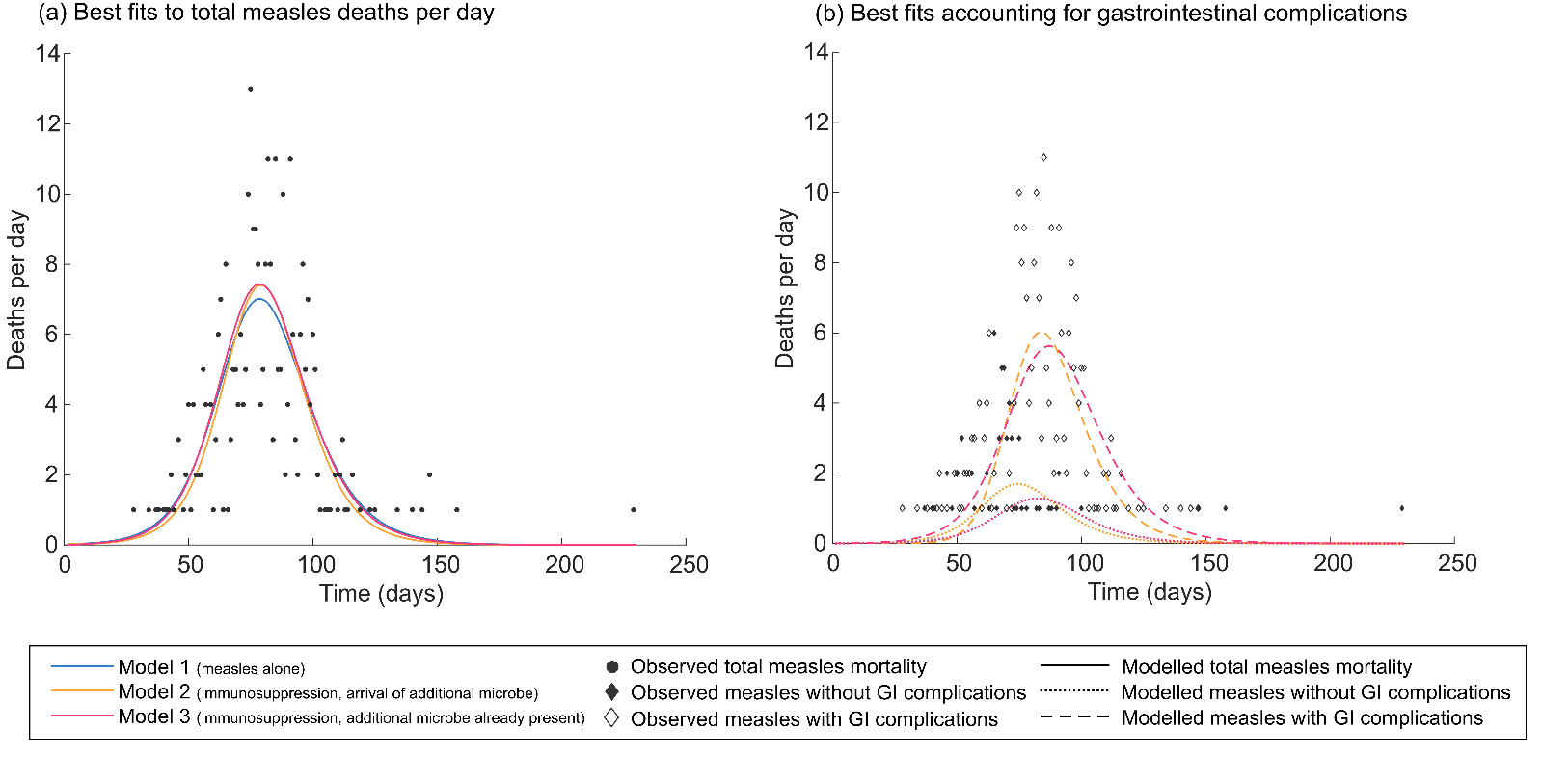
**

**Figure S2: Least squares fitting of mortality patterns during the 1911 measles outbreak on Rotuma.** Panel (a) illustrates the best fitting mortality time series generated by each of models 1-3 using least squares fitting when the models were fitted to the total number of measles deaths per day. Panel (b) illustrates the best fitting mortality time series for models 2 and 3 using least squares fitting when the models were fitted to the pattern of measles deaths with and without gastrointestinal complications. In both panels (a) and (b) the two individuals who brought measles to Rotuma were assumed to be in the infectious class.

**
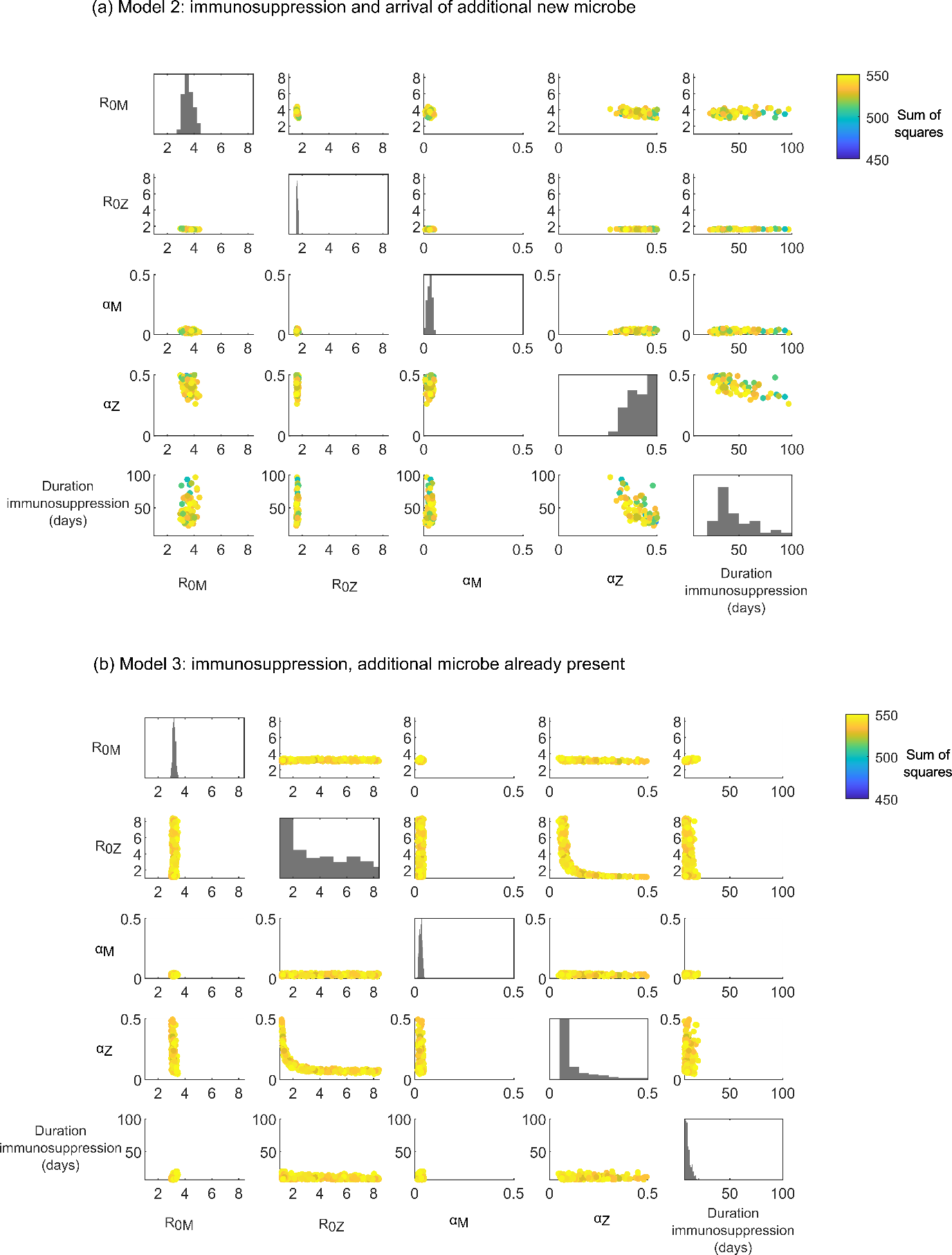
**

**Figure S3: Least squares fitting where those bringing measles to Rotuma are assumed to be in the infectious class at time 0.** This figure illustrates the distribution of parameter values associated with an acceptable fit (sum of squares <550) in our least squares analysis of model 2 (panel a) or model 3 (panel b), for starting conditions where we assume that the two initial measles infections on Rotuma were already infectious at time 0. The range of the x or y axis for each sub panel indicates the range of values tested for the labelled quantity. The parameters varied were: β_M_ , β_Z_ , α_M_  , α_Z_ and ω (see Table 1 for definitions). For ease of interpretation, parameters β_M_  and β_Z_ have been converted into R_0_ values and the inverse of parameter ω is shown (i.e. the duration of immunosuppression). All possible pairwise combinations of the parameters varied are shown as scatter plots (where the marker colour indicates the sum of squares associated with that combination of parameter values). We also show histograms of each parameter individually.

**
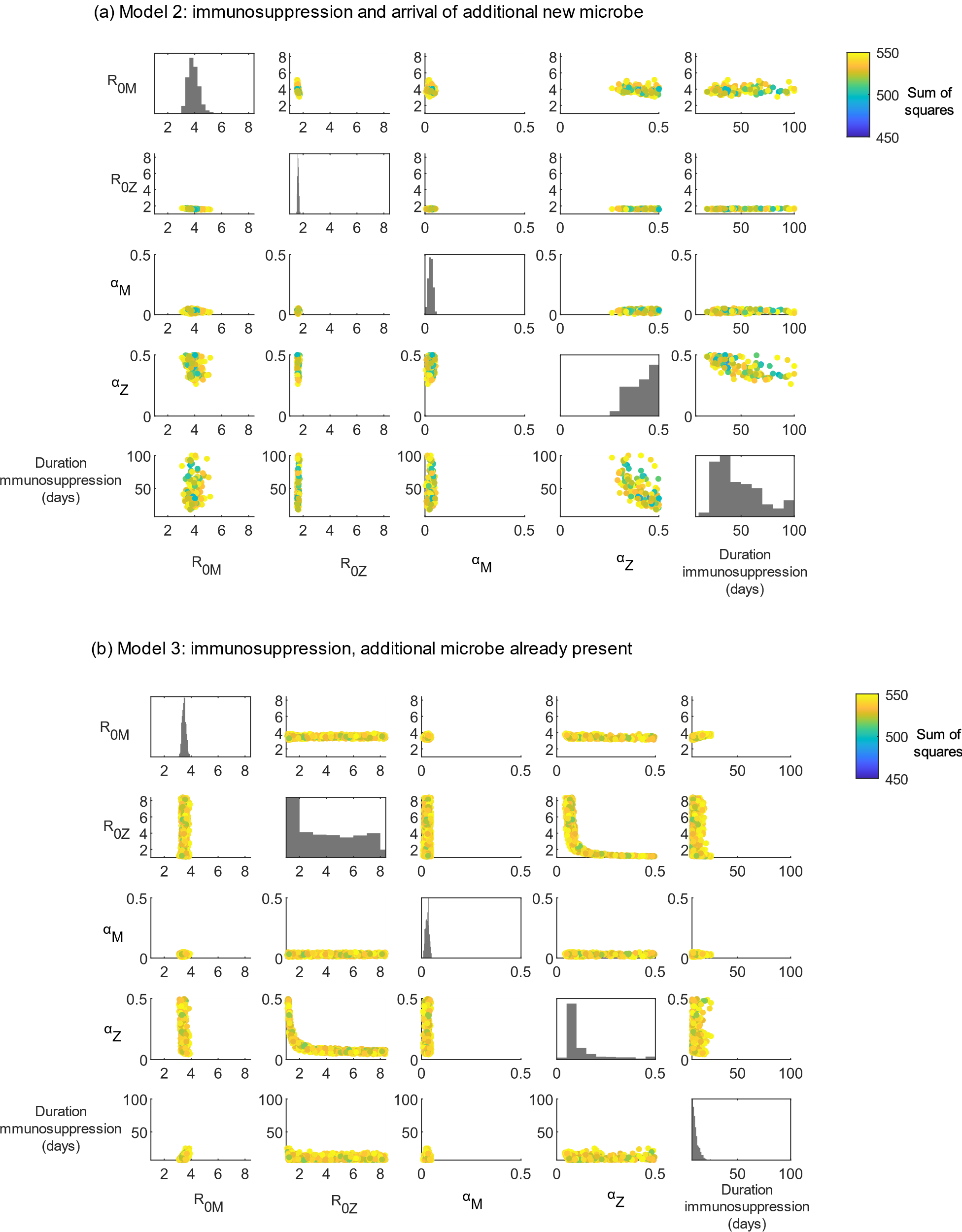
**

**Figure S4: Least squares fitting where those bringing measles to Rotuma are assumed to be in the exposed class at time 0.** This figure illustrates the distribution of parameter values associated with an acceptable fit (sum of squares <550) in our least squares analysis of model 2 (panel a) or model 3 (panel b), for starting conditions where we assume that the two initial measles infections on Rotuma were not yet infectious (i.e. in the “exposed” measles class) at time 0. The range of the x or y axis for each sub panel indicates the range of values tested for the labelled quantity. The parameters varied were: β_M_ , β_Z_ , α_M_  , α_Z_ and ω (see Table 1 for definitions). For ease of interpretation, parameters β_M_  and β_Z_ have been converted into R_0_ values and the inverse of parameter ω is shown (i.e. the duration of immunosuppression). All possible pairwise combinations of the parameters varied are shown as scatter plots (where the marker colour indicates the sum of squares associated with that combination of parameter values). We also show histograms of each parameter individually.

**
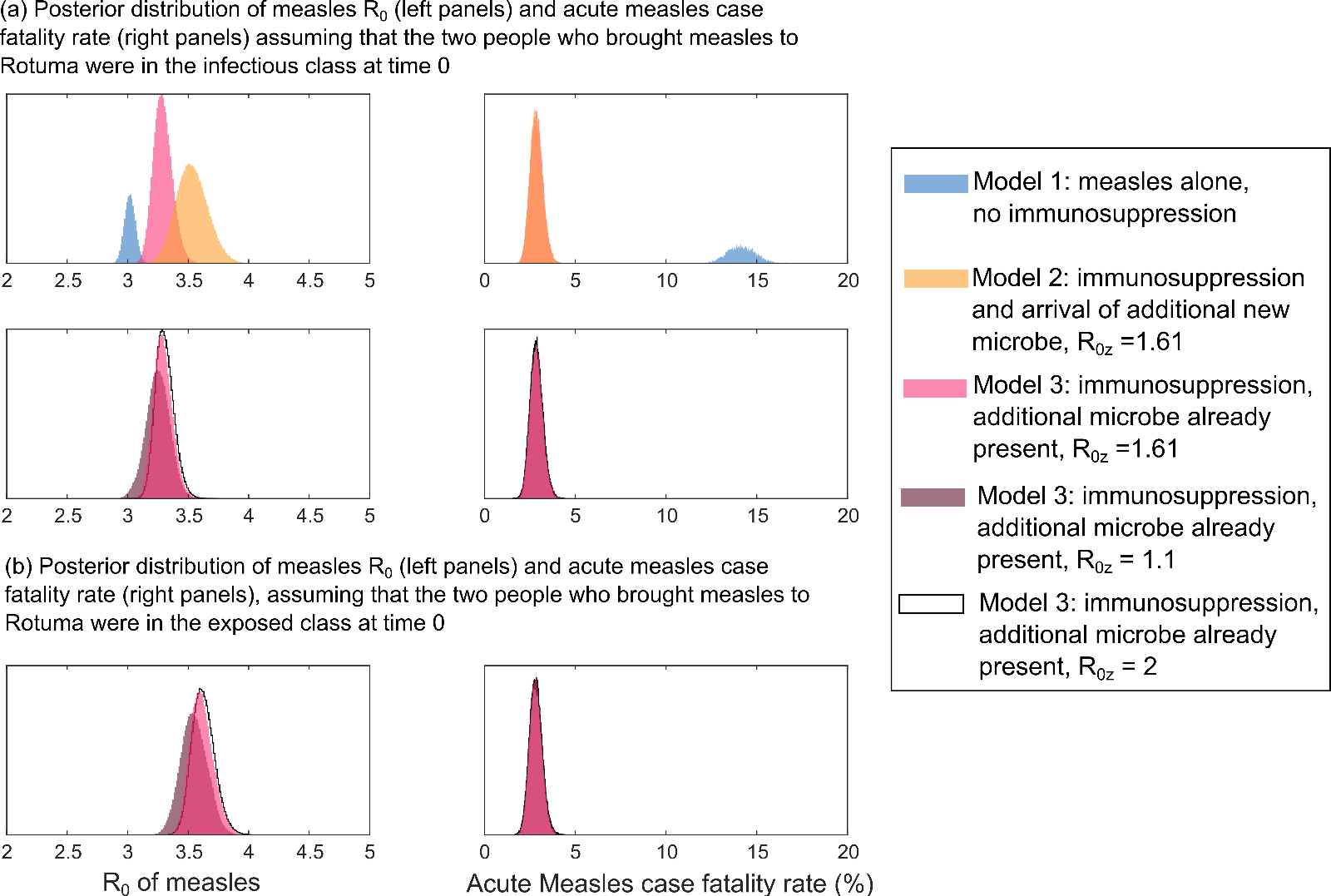
**

**Figure S5: Sensitivity analysis of measles R_0_ and acute measles case fatality rate results.** Figure 3 in the main text illustrates posterior distributions for measles R_0_ and the case fatality rate for acute measles infection, obtained for each of our 3 models when the individuals who brought measles to Rotuma were in the exposed class. We also fixed the value of R_0_ for the secondary infectious agent (R_0Z_) to be 1.61 because fixing this parameter made our MCMC analysis computationally feasible, and we knew that a value of R_0Z_ close to 1.61 was necessary for model 2 to fit the data (see figures S3 and S4). Here we show the effect of changing these assumptions. In (a), the individuals who brought measles to Rotuma are assumed to be in the infectious class. The top row of (a) compares all 3 models, as in figure 3 of the main text. The bottom row of (a) considers just model 3 and illustrates the impact of varying R_0Z_, because model 3 can fit the data with a variety of values of R_0Z_ (see figures S3 and S4). Panel (b) illustrates the impact of varying R_0Z_ on the posterior distribution of measles R_0_ and the acute measles case fatality rate generated by model 3, when the two individuals who brought measles to Rotuma are assumed to have been in the exposed class (just like in the main text).

**
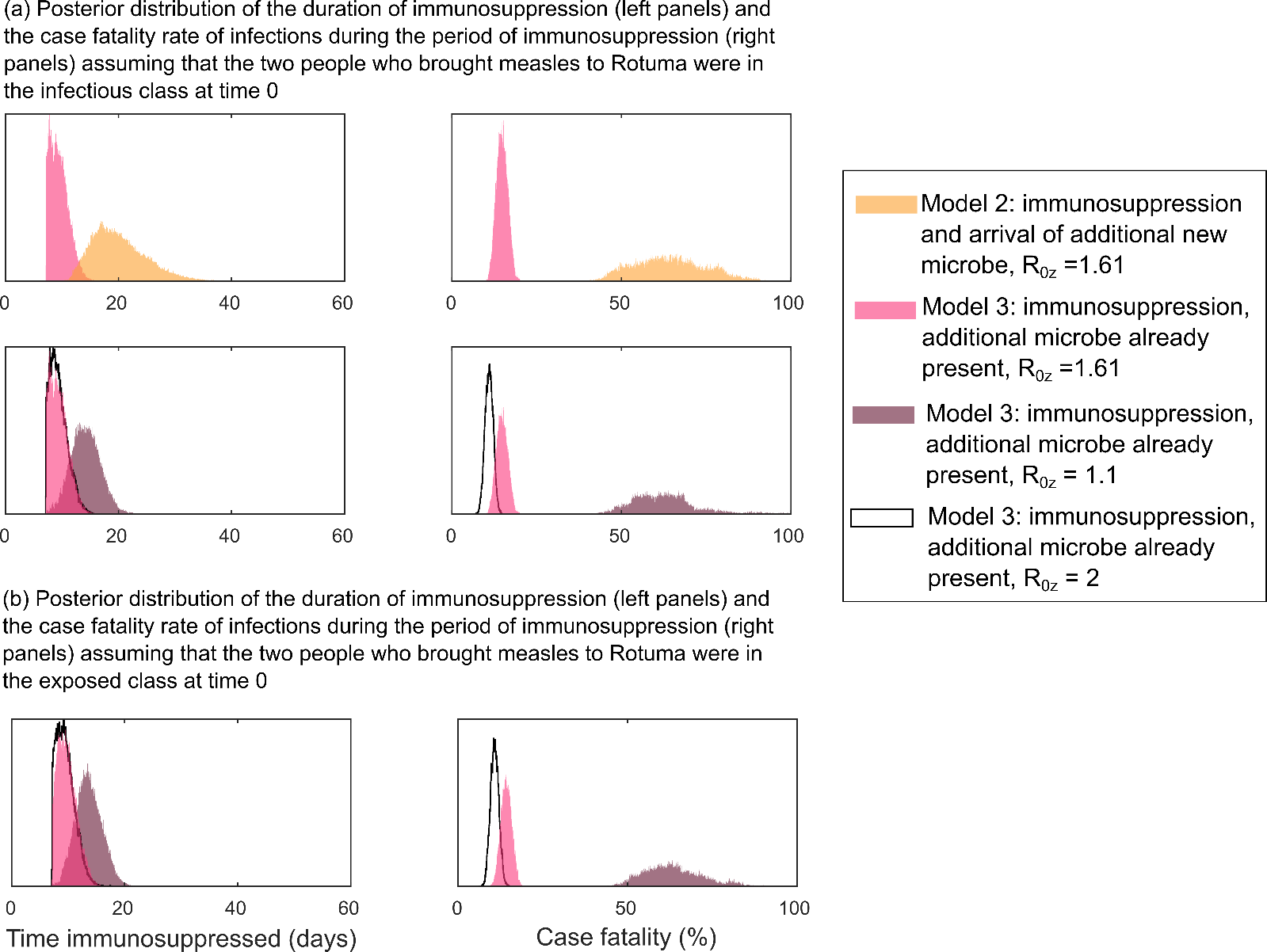
**

**Figure S6: Sensitivity analysis of immunosuppression duration and immunosuppressed case fatality rate results.** Figure 4 in the main text illustrates posterior distributions for the duration of immunosuppression and the case fatality rate for infections whilst immunosuppressed obtained for each of our 3 models when the individuals who brought measles to Rotuma were in the exposed class. We also fixed the value of R_0_ for the secondary infectious agent (R_0Z_) to be 1.61 because fixing this parameter made our MCMC analysis computationally feasible, and we knew that a value of R_0Z_ close to 1.61 was necessary for model 2 to fit the data (see figures S3 and S4). Here we show the effect of changing these assumptions. In (a), the individuals who brought measles to Rotuma are assumed to be in the infectious class. The top row of (a) compares all 3 models, as in figure 3 of the main text. The bottom row of (a) considers just model 3 and illustrates the impact of varying R_0Z_, because model 3 can fit the data with a variety of values of R_0Z_ (see figures S3 and S4). Panel (b) illustrates the impact of varying R_0Z_ on the posterior distribution of the duration of immunosuppression and the case fatality rate for infections whilst immunosuppressed generated by model 3, when the two individuals who brought measles to Rotuma are assumed to have been in the exposed class (just like in the main text).

**
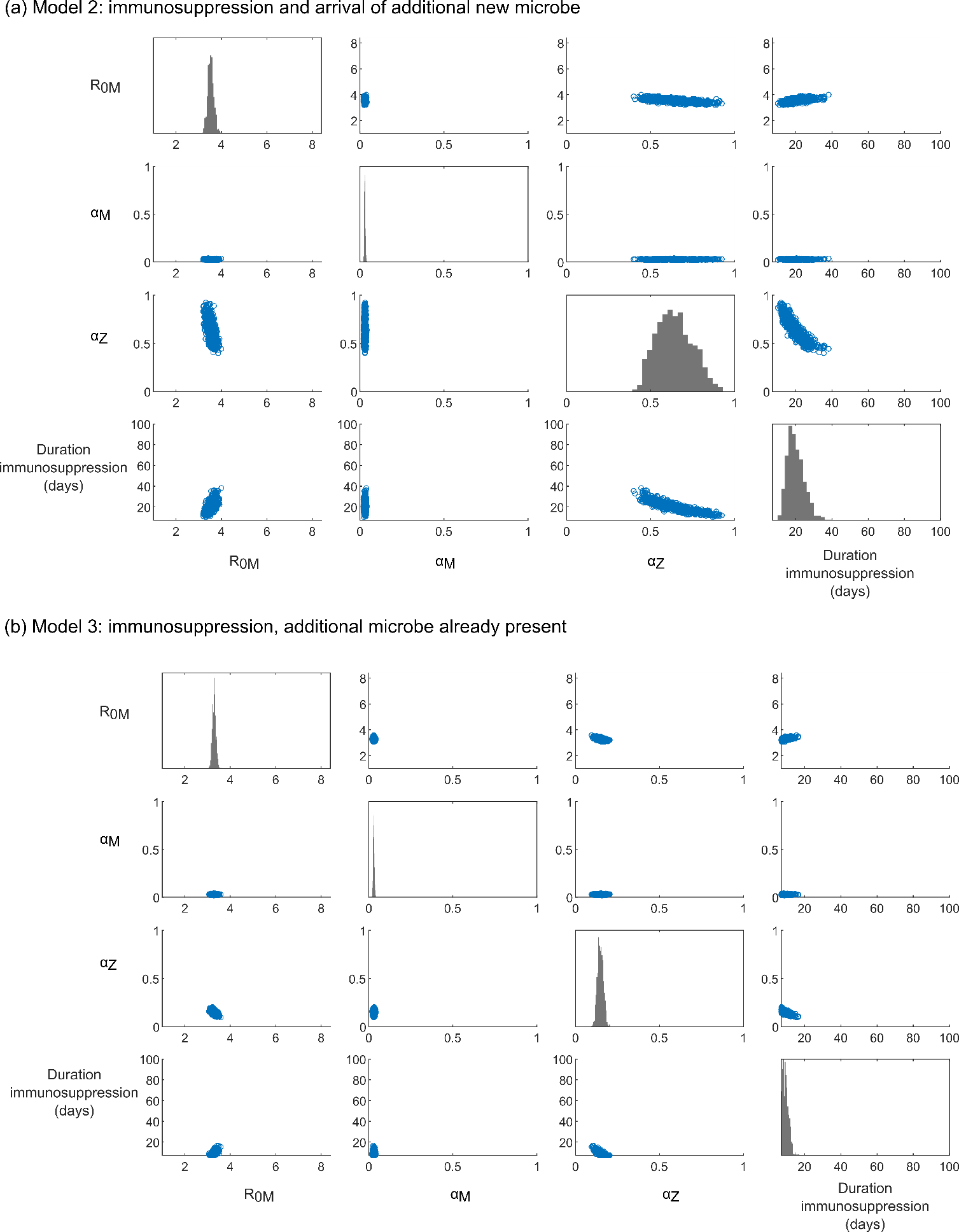
**

**Figure S7: MCMC fitting where those bringing measles to Rotuma are assumed to be in the infectious class at time 0.** This figure illustrates individual (histograms) and joint (scatter plots) posterior distributions obtained for the parameters we fitted in the MCMC analysis. The parameters fitted were: β_M_ , α_M_  , α_Z_ and ω (see Table 1 for definitions). For ease of interpretation, β_M_ has been converted into the R_0_ implied for measles by that value of β_M_ and the inverse of parameter ω is shown (i.e. the duration of immunosuppression). The prior distribution for each parameter was a uniform distribution with maximum and minimum values that are equal to the range of the x or y axis in each sub panel.

**
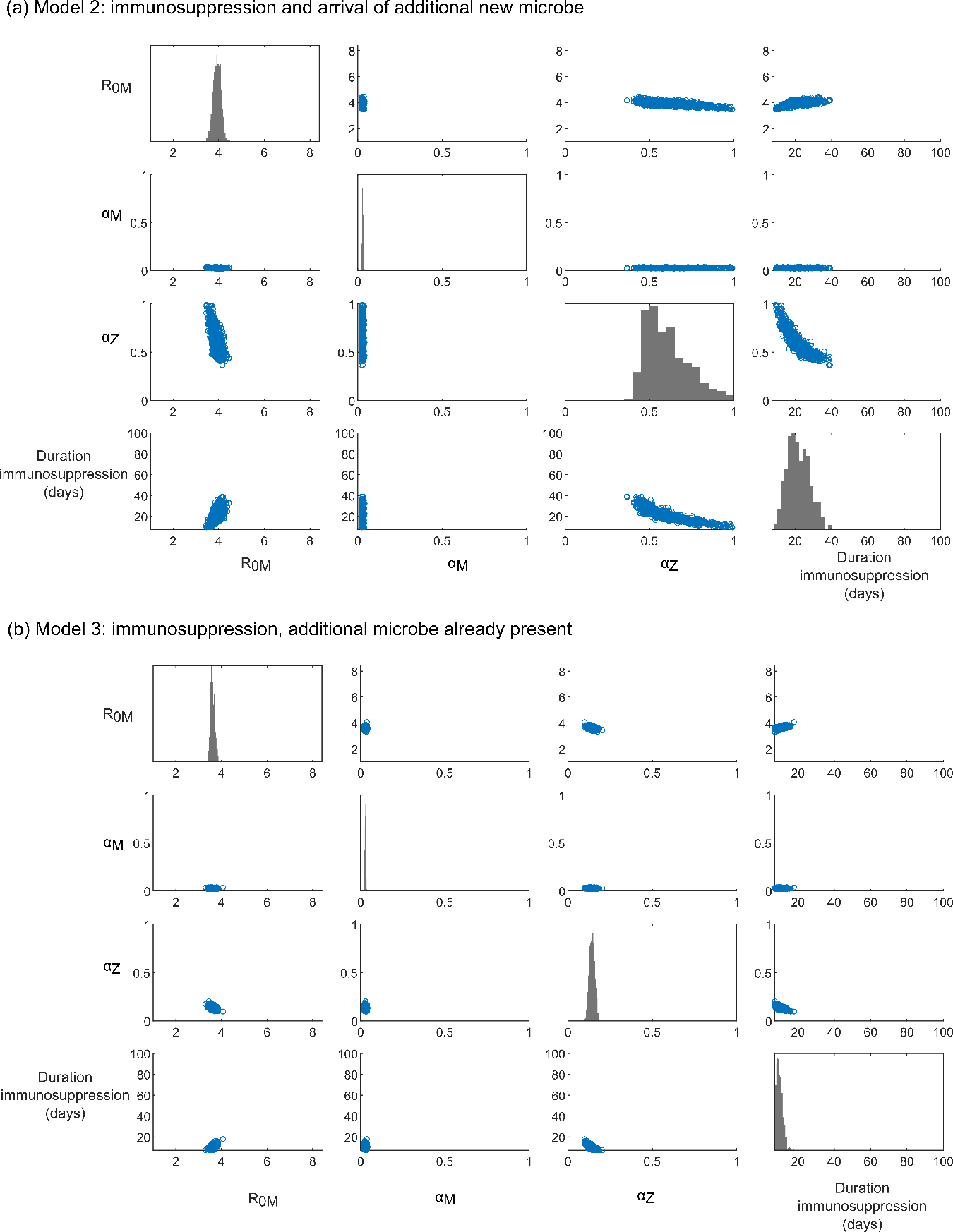
**

**Figure S8: MCMC fitting where those bringing measles to Rotuma are assumed to be in the exposed class at time 0.** This figure illustrates individual (histograms) and joint (scatter plots) posterior distributions obtained for the parameters we fitted in the MCMC analysis. The parameters fitted were: β_M_ , α_M_  , α_Z_ and ω (see Table 1 for definitions). For ease of interpretation, β_M_ has been converted into the R_0_ implied for measles by that value of β_M_ and the inverse of parameter ω is shown (i.e. the duration of immunosuppression). The prior distribution for each parameter was a uniform distribution with maximum and minimum values that are equal to the range of the x or y axis in each sub panel.
